# An Orthotopic Model of Uveal Melanoma in Zebrafish Embryo: A Novel Platform for Drug Evaluation

**DOI:** 10.3390/biomedicines9121873

**Published:** 2021-12-10

**Authors:** Chiara Tobia, Daniela Coltrini, Roberto Ronca, Alessandra Loda, Jessica Guerra, Elisa Scalvini, Francesco Semeraro, Sara Rezzola

**Affiliations:** 1 Department of Molecular and Translational Medicine, University of Brescia, 25123 Brescia, Italy; chiara.tobia@unibs.it (C.T.); daniela.coltrini@unibs.it (D.C.); roberto.ronca@unibs.it (R.R.); a.loda025@unibs.it (A.L.); jessica.guerra@unibs.it (J.G.); scalvini.elisa@yahoo.it (E.S.); 2 Eye Clinic, Department of Medical and Surgical Specialties, Radiological Sciences and Public Health, University of Brescia, 25123 Brescia, Italy; francesco.semeraro@unibs.it

**Keywords:** uveal melanoma, zebrafish, orthotopic tumor, xenograft, luciferase

## Abstract

Uveal melanoma is a highly metastatic tumor, representing the most common primary intraocular malignancy in adults. Tumor cell xenografts in zebrafish embryos may provide the opportunity to study in vivo different aspects of the neoplastic disease and its response to therapy. Here, we established an orthotopic model of uveal melanoma in zebrafish by injecting highly metastatic murine B16-BL6 and B16-LS9 melanoma cells, human A375M melanoma cells, and human 92.1 uveal melanoma cells into the eye of zebrafish embryos in the proximity of the developing choroidal vasculature. Immunohistochemical and immunofluorescence analyses showed that melanoma cells proliferate during the first four days after injection and move towards the eye surface. Moreover, bioluminescence analysis of luciferase-expressing human 92.1 uveal melanoma cells allowed the quantitative assessment of the antitumor activity exerted by the canonical chemotherapeutic drugs paclitaxel, panobinostat, and everolimus after their injection into the grafted eye. Altogether, our data demonstrate that the zebrafish embryo eye is a permissive environment for the growth of invasive cutaneous and uveal melanoma cells. In addition, we have established a new luciferase-based in vivo orthotopic model that allows the quantification of human uveal melanoma cells engrafted in the zebrafish embryo eye, and which may represent a suitable tool for the screening of novel drug candidates for uveal melanoma therapy.

## 1. Introduction

The zebrafish embryo has been successfully employed as a platform for modeling human diseases and for large-scale screening of new drugs [1,2,3,4]. Ease of manipulation, relatively low costs of maintenance, and optical transparency, combined with the opportunity to perform high-quality imaging, led to an extensive use of this model in cancer research. In this regard, mammalian tumor cell grafting in zebrafish embryos can be achieved in different anatomical sites, giving opportunity to study various aspects of the disease, such as tumor progression, angiogenesis, cancer cell spreading, and metastasis formation. Tumor cells have been successfully implanted in the perivitelline space, yolk ball, blood stream, pericardial cavity, eye, and brain (see [5,6,7,8,9] and references therein).

One of the major drawbacks of the use of the zebrafish embryo as a model in oncology is the quantification of tumor xenograft growth in the different anatomical sites, generally performed by measuring the fluorescence signal generated by engrafted fluorescent tumor cells [10,11]. This approach has also been used for the study of cancer growth following ocular transplantation of fluorescent tumor cells in zebrafish embryos [7,8,9]. However, the presence of the lens and the cup-like structure of the eye make difficult the acquisition of high-quality fluorescence images, which may lead to misleading results. This calls for alternative rapid and reliable quantification methods to be exploited for high throughput analysis.

Uveal melanoma represents the most common primary intraocular malignancy in adults. Classified as a rare neoplasm, its occurrence increases with age and its incidence is over 20 million/year. Despite the results obtained in terms of primary tumor management, the 5-year mortality rate of uveal melanoma patients (ranging from 26 to 32%) has not changed over the years [12,13,14,15]. Indeed, almost 50% of uveal melanoma patients develop metastatic disease through haematogenous dissemination [16], leading to an approximately 5–7-month median survival [13,14] which is rarely improved by chemotherapy [17]. At present, no drugs have been approved for the treatment of metastatic uveal melanoma patients and new therapeutic strategies are eagerly required. Nevertheless, despite the urgent need for an in vivo platform for the rapid screening of novel drug candidates, an orthotopic uveal melanoma model has not yet been implemented with zebrafish embryos.

Here, we propose a luciferase-based quantification method to demonstrate that transplantation of uveal melanoma cells into the eye of zebrafish embryos represents a useful in vivo orthotopic model suitable for the screening of novel drug candidates for uveal melanoma therapy.

## 2. Materials and Methods

### 2.1. Reagents

All reagents were of analytical grade. Dulbecco’s modified Eagle medium (DMEM), RPMI 1640 medium, fetal bovine serum (FBS), non-essential amino acid (NEAA), and MEM vitamin solutions were obtained from GIBCO Life Technologies (Grand Island, NY, USA). Penicillin, streptomycin, sodium pyruvate, PTU, tricaine, bovine serum albumin (BSA), diaminobenzydine (DAB), and mouse anti-mouse vimentin antibody (Vim 13.2 clone) were from Sigma-Aldrich (St. Louis, MO, USA). Paclitaxel, panobinostat, and everolimus were from MedChemExpress (Monmouth Junction, NJ, USA). The Annexin-V/propidium iodide double staining kit was from Immunostep Biotec (Salamanca, Spain). The ONE-Glo™ Luciferase Assay System was from Promega (Milan, Italy). Rat anti-mouse Ki-67 antibody (TEC-3) was from Dako (Santa Clara, CA, USA). Rabbit anti-human cleaved caspase 3 (Asp175) was from Cell Signaling (Danvers, MA, USA). Biotinylated anti-mouse IgM, anti-rat, and rabbit antibodies were from Abcam (Cambridge, UK). Biotin Avidin system Vectastain ABC reagent was from Vector Laboratories (Burlingame, CA, USA).

### 2.2. Cell Cultures

Murine melanoma B16-BL6 cells were grown in DMEM plus 10% FBS and 1% penicillin/streptomycin, and were stably transfected with DsRed fluorescent protein, thus generating B16-BL6-DsRed^+^ cells [2]. Murine melanoma B16-LS9 cells [18] were kindly provided by Dr. L. Morbidelli (University of Siena, Siena, Italy) and were grown in DMEM plus 10% FBS, 1% penicillin/streptomycin. Luciferase-transfected B16-LS9 cells (B16-LS9-luc^+^ cells) were generated as previously described [19]. Human melanoma A375M cells were obtained from Dr. R. Giavazzi (Istituto Ricerche Farmacologiche Mario Negri, Bergamo, Italy) and were grown in DMEM plus 20% FBS, 1% NEAA, 2% MEM vitamin solution, 1% sodium pyruvate, and 1% penicillin/streptomycin. Human uveal melanoma 92.1 cells [20] were obtained from Dr. M. Jager (Leiden University, Leiden, The Netherlands) and were maintained in RPMI 1640 medium plus 10% FBS, 1% penicillin/streptomycin. A375M and 92.1 cells were infected with a lentivirus harboring the RFP/luciferase cDNA, thus generating stable A375M-RFP^+^/luc^+^ and 92.1-RFP^+^/luc^+^ cells that express both the red fluorescent RFP protein and the bioluminescent firefly luciferase. For eye injection, cells were suspended in PBS (final concentration equal to 100,000 cells/µL).

### 2.3. Cell Proliferation Assay

Cells were seeded on 48-well plates at 1.0 × 10^4^ cells/cm^2^ or at 1.5 × 10^4^ cells/cm^2^ for B16-LS9-luc^+^ and 92.1-RFP^+^/luc^+^ cells, respectively. After 24 h, cells were treated with increasing concentrations of the different anticancer drugs. After a further 48 h or 72 h incubation, cells were trypsinized and viable cell counting was performed with the MACSQuant^®^ Analyzer (Miltenyi Biotec, Bergisch Gladabach, Germany), as reported [21].

### 2.4. Apoptosis Assay

92.1-RFP^+^/luc^+^ cells were seeded on 6-well plates at 1.0 × 10^4^ cells/cm^2^. After 24 h, cells were treated with 140 nM paclitaxel, 20 nM panobinostat, and 60 nM everolimus. After 72 h of treatment, apoptotic cell death was assessed by Annexin-V/propidium iodide double staining according to the manufacturer’s instructions, and cytofluorimetric analysis was performed using the MACSQuant^®^ Analyzer.

### 2.5. Zebrafish Maintenance and Cell Transplantation

The transgenic tg(*kdrl*:EGFP) zebrafish line was maintained in the facility of the University of Brescia at 28 °C under standard conditions [22], and embryos were staged by h post-fertilization (hpf), as described [23]. To prevent pigmentation, embryo fish water was added with 0.2 mM 1-phenyl-2-thiourea (PTU) starting from 24 hpf. For cell injection and in vivo observation, embryos were anesthetized using 0.16 mg/mL tricaine. For cell engrafting, 48 hpf embryos were microinjected in the eye with tumor cells using a borosilicate needle and an Eppendorf FemtoJet microinjector equipped with an InjectMan NI2 manipulator. A single eye was injected with tumor cells in each zebrafish embryo. When indicated, 2.0 nL of a solution containing the anticancer drug under testing was injected in the same eye. After tumor cell injection, zebrafish embryos were selected under a fluorescence microscope to ensure that tumor cells were located only within the eyeballs and then grown at 33 °C.

### 2.6. Fluorescence and Light Sheet Microscopy

Live embryos were photographed at 1 h (t_0_, 48 hpf), 1 day (t_1_) and 4 days (t_4_) post implantation on agarose-coated dishes using an AxioZoom V16 fluorescence stereomicroscope (Zeiss, Oberkochen, Germany, EU) equipped with a digital Axiocam 506 color camera (Zeiss). The mean area of the tumor was manually measured using FIJI software [24]. Light sheet microscopy experiments were performed using a Light Sheet Z.1 microscope (Zeiss). For this purpose, t_0_, t_1_, and t_4_ embryos were embedded in a low melting agarose cylinder (1% low melting agarose:fish water, 1:1) and immersed in the observation chamber filled with fish water and anesthetic. Maximum intensity projections were obtained using the Zen software (Zeiss) and 3D reconstructions were made after z-stack processing with Arivis software (Zeiss).

To detect apoptotic cells, 48 hpf embryos were microinjected in the eye with 2.0 nL of a solution containing the anticancer drug under testing. After injection, zebrafish embryos were grown at 33 °C for 4 days. At t_4_, live embryos were soaked in fish water containing 2 μg/mL acridine orange and incubated at 28 °C for 20 min. After 8 washes for 5 min each with fish water, embryos were anesthetized and analyzed immediately with a fluorescence stereomicroscope (Zeiss).

### 2.7. Immunohistochemical Analysis

After tumor cell injection, zebrafish embryos were formalin-fixed, paraffin-embedded, and sections of grafted eyes were analyzed at t_0_ and t_4_ by hematoxylin and eosin (H&E) or immunohistochemical staining [25]. Briefly, sections were de-waxed, rehydrated, and endogenous peroxidase activity blocked with 0.3% H_2_O_2_ in methanol. Antigen retrieval was performed using a thermostatic bath (Labochema, Vilnius, Lithuania), in 10 mM citrate buffer (pH 6.0). Sections were then washed in TBS (pH 7.4) and incubated overnight with a mouse monoclonal (IgM isotype) anti-mouse vimentin antibody (1:200) or with a rat anti-mouse Ki-67 antibody (1:100) or with a rabbit anti-human cleaved caspase 3 (Asp175) (1:100) diluted in TBS plus 1% BSA, 0.1% Triton x-100, and 0.1% Tween, followed by 1 h incubation with biotinylated anti-mouse IgM, anti-rat, or anti-rabbit antibody (1:200), respectively. Signal was revealed using Biotin Avidin system Vectastain ABC reagent followed by DAB as chromogen and hematoxylin as counterstain. Images were taken using an Axio Imager A2 microscope equipped with a digital AxioCam MRc5 camera (Zeiss).

### 2.8. Luciferase-Based Quantification Method

At different time points after intraocular grafting of luc^+^ cells, enucleated eyes or anesthetized embryos were singularly placed in a well of a white polystyrene 96-well plate (Sigma-Aldrich). Embryo medium was removed and replaced with 50 µL of lysis buffer (80 mM Na_2_HPO_4_, 9.3 mM NaH_2_PO_4_, 2% TritonX100, 1.0 mM DTT in MilliQ water) and 50 µL of ONE-Glo™ Reagent. The luminescence was measured using an EnSight^®^ Multimode Plate Reader (PerkinElmer, Milan, Italy) and expressed as relative luminescence units (RLUs).

To generate the calibration curve, a fixed number of B16-LS9-luc^+^ cells (ranging from 0 to 1000 cells) was added to non-injected embryos and then the bioluminescence signal quantified as described above.

### 2.9. Statistical Analysis

Statistical analysis was performed with GraphPad Prism 8 (San Diego, CA, USA) using a Student’s *t*-test for 2 groups of samples or one-way analysis of variance followed by Tukey’s multiple comparison post hoc test for more than 2 groups. Differences were considered significant when *p*-values < 0.05.

## 3. Results and Discussion

### 3.1. Zebrafish Embryo Eye Is a Permissive Environment for the Growth of Engrafted Melanoma Cells

**Figure 1:**
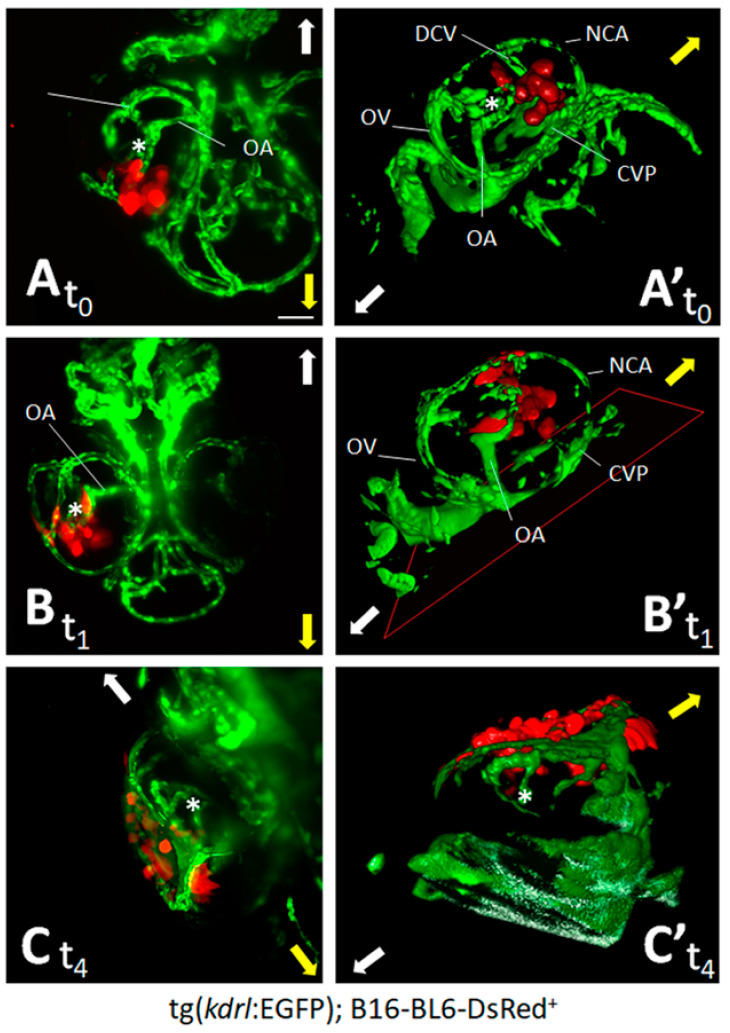
Zebrafish embryo eye is a microenvironment suitable for cell grafting. Murine melanoma B16-BL6-DsRed^+^ cells (100 cells/embryo) were orthotopically injected in the posterior side of the developing eye of transgenic tg(*kdrl*:EGFP) zebrafish embryos at 48 hpf. Maximum intensity projection of the z-stacks (**A**–**C**) and 3D reconstructions (**A’**–**C’**) of B16-BL6-DsRed^+^ cells performed at 1 h (t_0_) (**A**,**A’**), 1 day (t_1_) (**B**,**B’**), and 4 days (t_4_) (**C**,**C’**) post implantation. (**A**,**B**) ventral view; (**C**) dorsal view. Asterisk indicates the hyaloid artery. Arrows indicate embryo orientation: white arrow, posterior side; yellow arrow, anterior side. CVP, choroidal vascular plexus; DCV, dorsal ciliary vein; NCA, nasal ciliary artery; OA, optic artery; OV, optic vein. Scale bar: 50 µm.

**Figure 2:**
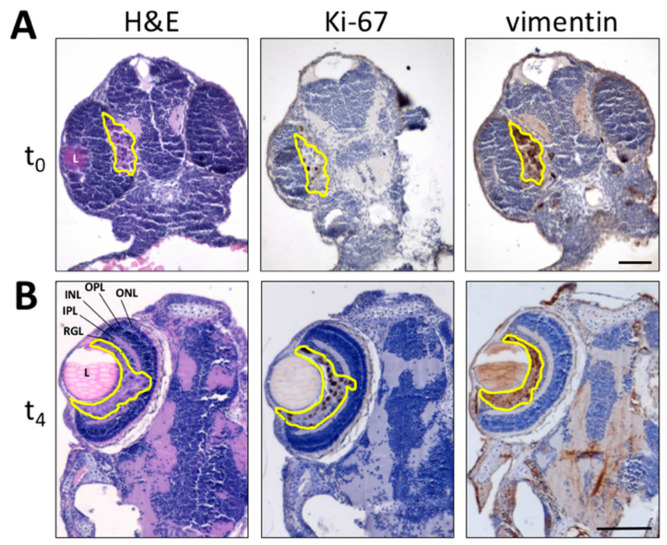
Histological analysis of melanoma B16-BL6-DsRed^+^ xenografts. Paraffin sections of B16-BL6-DsRed^+^ cells grafted into zebrafish embryo eyes obtained at 1 h (t_0_) (**A**) or 4 days (t_4_) post implantation (**B**) are stained by H&E (**left panel**) whereas Ki-67 (**central panel**) and vimentin (**right panel**) immunoreactivity is shown in brown. Tumor area is highlighted in yellow. L, lens; INL, inner nuclear layer; IPL, inner plexiform layer; ONL, outer nuclear layer; OPL, outer plexiform layer; RGL, retinal ganglion cell layer. Scale bars: 50 µm.

To evaluate whether the zebrafish embryo eye represents a microenvironment suitable for the grafting of melanoma cells, we first assessed the behavior of the well-characterized model of invasive murine melanoma represented by B16-BL6-DsRed^+^ cells [2], which were injected into the eye of zebrafish embryos at 48 hpf. At this stage, the embryo eye consists of the retina (mainly composed of neuronal cells that will progressively organize in stratified retinal layers [26]), the hyaloid, and the ciliary vascular systems [27]. On this basis, B16-BL6-DsRed^+^ cells were orthotopically injected in the posterior side of the developing eye of tg(*kdrl*:EGFP) embryos (100 cells/embryo) and monitored for the following 4 days by light sheet fluorescence microscopy. One hour after implantation (t_0_), maximum intensity projection of the z-stacks and 3D reconstructions confirmed that DsRed^+^ cells were present at the bottom of the eye in the proximity of the developing choroidal vasculature (Figure 1A,A’). One day post implantation (t_1_), cells relocate towards the eye surface, interacting with the surrounding vasculature (Figure 1B,B’). At 4 days post implantation (t_4_), DsRed^+^ cells invaded the lens surface and grew without exerting a significant impact on the anatomical architecture of the eye (Figure 1C,C’). To confirm these observations, paraffin sections of tumor cell-grafted eyes were analyzed at t_0_ and t_4_ by H&E staining and by Ki-67 and vimentin immunostaining. As shown in Figure 2, implanted B16-BL6-DsRed^+^ cells were able to proliferate, as demonstrated by the presence of Ki-67^+^ cells, without affecting the physiological development of the retina. Moreover, cells moved towards the eye surface and invaded the lens (Figure 2B). Notably, preliminary observations suggest that the displacement of melanoma cells observed at t_4_ is in part a consequence of the invasive properties of cancer cells and in part due to the remodeling of the eye that occurs during embryo development, which plays a not negligible role in tumor cell localization within the eye (data not shown).

### 3.2. Quantification of Melanoma Xenograft Growth in the Zebrafish Embryo Eye

To obtain a reliable and reproducible quantification of melanoma cell growth in zebrafish embryo eyes, we performed a first set of experiments exploiting the fluorescence signal of B16-BL6-DsRed^+^ cells. For this purpose, we attempted to measure fluorescent tumor areas in engrafted embryos at t_0_, t_1_, and t_4_ after injection. As anticipated, even though the analysis of digitalized images demonstrated an increase of DsRed^+^ tumor areas at t_4_ when compared to the other time points (Supplementary Materials, Figure S1), the results suffered significant drawbacks. Indeed, although extended depth of focus of the z-stacks provided a good quality lateral view of the xenografts at t_0_ and t_1_, the acquisition of images required to cover the entire thickness of the tumor was difficult at t_4_ and was affected by the position of tumor cells that were close to the lens or deeply immersed in the eye.

In addition, the three-dimensional structure of the embryo eye and the presence of the lens, which may generate distorted images, made problematic the choice of the best angle for image acquisition. In this context, the optical accessibility of the zebrafish eye is further limited by the presence of pigmented cells, including neural crest-derived chromatophores (i.e., melanophores, iridophores, and xanthophores) and the retinal pigment epithelium [28]. Moreover, the blockade of zebrafish pigmentation by the addition of PTU in the fish water [22] has no effect on iridophores and on their nonspecific fluorescence signal [29], which impairs the reproducibility of the quantification technique.

On the other hand, it has been shown that the use of transparent *crystal* zebrafish mutants does not completely avoid refraction of the light due to the presence of the lens and of residual xanthophores present in the mutant eyes [29]. Finally, even though high-quality images may be obtained by confocal microscopy [11], acquisition and analysis procedures are time consuming and not suitable for high-throughput analysis.

To overcome these limitations, we developed an alternative quantification method exploiting the bioluminescence signal generated by tumor cells transduced with firefly luciferase. To this end, we took advantage of a red fluorescent and luciferase expressing human melanoma cell line (A375M-RFP^+^/luc^+^) available in our laboratory. A375M-RFP^+^/luc^+^ cells were grafted in the eye of 48 hpf-old zebrafish embryos at 50, 100, and 200 cells/injection. Then, injected and not injected contralateral eyes were enucleated 1 h after grafting. As shown in the Supplementary Materials, Figure S2, analysis of grafted eyes indicates that the bioluminescence signal increases in a cell dose-dependent manner, being distinct from the basal levels measured in the contralateral control eyes. Similar results were obtained by measuring the bioluminescence signal generated by the lysates of the whole embryos engrafted with A375M-RFP^+^/luc^+^ cells (data not shown), thus avoiding the technically difficulty and the time-consuming eye enucleation procedure and confirming the reliability of this quantification method.

**Figure 3:**
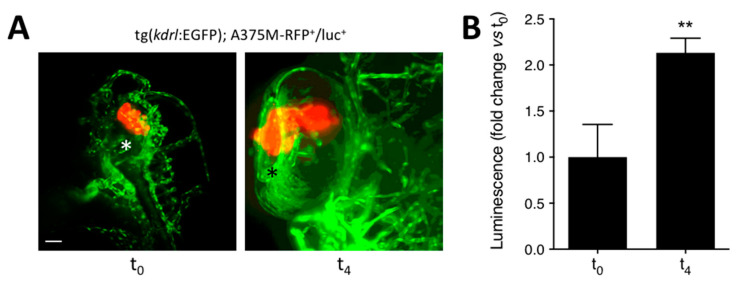
Luciferase-based quantification of the growth of human melanoma A375M-RFP^+^/luc^+^ xenografts. Human melanoma A375M-RFP^+^/luc^+^ cells (100 cells/embryo) were injected into the posterior side of the developing eye of transgenic tg(*kdrl*:EGFP) zebrafish embryos at 48 hpf. (**A**) Maximum intensity projection of the z-stacks of A375M-RFP^+^/luc^+^ cells performed at 1 h (t_0_) and 4 days (t_4_) post implantation. T_0_, lateral view, anterior to the top; t_4_, dorsal view, anterior to the top. Asterisk indicates the superficial ocular vasculature. Scale bar: 50 µm. (**B**) Evaluation of A375M-RFP^+^/luc^+^ bioluminescence signal in the lysates of the whole embryos at t_0_ and t_4_. Data are the mean ± SEM (*n* = 8). ** *p* < 0.01 vs. t_0_, Student’s *t*-test.

To assess whether this procedure allowed a quantitative evaluation of the growth of grafted tumors, A375M-RFP^+^/luc^+^ cells (100 cells/embryo) were injected in the eye of tg(*kdrl*:EGFP) embryos at 48 hpf. Then, injected embryos were analyzed at t_0_ and t_4_ by light sheet fluorescence microscopy or by evaluation of the bioluminescence of the lysates of the whole animals. As shown in Figure 3A, A375M-RFP^+^/luc^+^ cells were clearly visible 1 h after grafting in the embryo eye. At 4 days post implantation, grafted cells were alive and had moved from the injection site toward the lens surface, as already observed for B16-BL6-DsRed^+^ cells. In parallel, a significant increase of the A375M-RFP^+^/luc^+^ cell-related bioluminescence signal was measured at t_4_ when compared to t_0_, thus confirming the capacity of this protocol to monitor the relative growth of tumor grafts (Figure 3B).

### 3.3. Orthotopic Ocular Grafting in the Zebrafish Embryo as a Model for Uveal Melanoma Treatment

**Figure 4:**
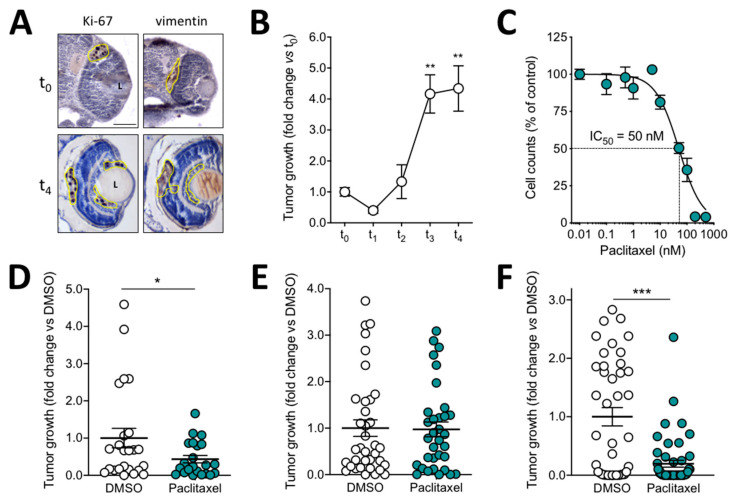
Effect of paclitaxel on the growth of murine melanoma B16-LS9-luc^+^ xenografts. (**A**) Immunohistochemical analysis of zebrafish embryo eyes at 1 h (t_0_) and 4 days (t_4_) after orthotopic injection of B16-LS9-luc^+^ cells. Ki-67 (**left panel**) and vimentin (**right panel**) are detected in brown. Tumor area is highlighted in yellow. L, lens. Scale bar: 50 µm. (**B**) B16-LS9-luc^+^ bioluminescence signal was evaluated 1 h (t_0_), 1 day (t_1_), 2 days (t_2_), 3 days (t_3_), and 4 days (t_4_) post implantation in the lysates of the whole embryos. Data are the mean ± SEM of five independent experiments. ** *p* < 0.01 vs. t_0_ and t_1_, ANOVA. (**C**) Effect of paclitaxel on the proliferation of B16-LS9-luc^+^ cells in vitro. Viable cells were counted after 72 h of incubation with increasing concentrations of the drug. Data are the mean ± SEM of three independent experiments. (**D**) B16-LS9-luc^+^ cells were cultured for 24 h in vitro in the absence or in the presence of 0.5 µM paclitaxel or with the corresponding volume of DMSO and then grafted in the zebrafish eye. Tumor growth was evaluated at t_4_ by measuring the cell luminescence signal in the lysates of the whole embryos. Data are the mean ± SEM (*n* = 20). * *p* < 0.05 vs. DMSO, Student’s *t*-test. (**E**) After injection of B16-LS9-luc^+^ cells into the zebrafish eye, embryos were incubated at t_0_ with 10 µM paclitaxel or with the corresponding volume of DMSO, both dissolved in fish water. Tumor growth was evaluated at t_4_ by measuring the cell luminescence signal. Data are the mean ± SEM (*n* = 35). (**F**) After B16-LS9-luc^+^ cell grafting into the zebrafish eye, 0.4 pmoles/embryo of paclitaxel or of the corresponding volume of DMSO were injected in the same eye. Tumor growth was evaluated at t_4_ by measuring the cell luminescence signal. Data are the mean ± SEM (*n* = 45). In (**D**–**F**), each dot represents one embryo. *** *p* < 0.0001 vs. DMSO, Student’s *t*-test.

Given the promising capacity of luciferase-expressing melanoma cells to grow and to be quantified after grafting in zebrafish eyes, we decided to extend this assay to a well-established murine melanoma model suitable for investigating the mechanisms responsible for uveal melanoma liver tropism [30,31,32], immunologic and angiogenic aspects [33], and drug response [34,35,36,37]. On this basis, B16-LS9-luc^+^ cells were injected in the zebrafish embryo eye, grafts were analyzed at t_0_ and t_4_, and immunohistochemical analysis of cell grafts showed that tumor cells proliferate, as already observed for B16-BL6 tumors (Figure 4A). In addition, bioluminescence quantification performed at different time points after injection (t_0_, t_1_, t_2_, t_3_, and t_4_) showed that, after a slight decrease in cell growth at t_1_, B16-LS9-luc^+^ cells proliferate rapidly, their cell number increasing up to four times at t_3_/t_4_ when compared to t_0_ (Figure 4B and Supplementary Materials, Figure S3).

In order to assess the response of tumor cells grafted in the embryo eye to anticancer drugs, preliminary experiments were carried out in which B16-LS9-luc^+^ cells were treated in vitro for 72 h with increasing concentrations of the microtubule-disrupting agent paclitaxel [38]. As shown in Figure 4C, the compound inhibits the growth of B16-LS9-luc^+^ cells with an ID_50_ equal to 50 nM. On this basis, three different routes of in vivo administration of the drug were attempted in engrafted zebrafish embryos: (i) 24 h in vitro pretreatment of B16-LS9-luc^+^ cells with 0.5 µM paclitaxel, followed by their injection in the zebrafish eye; (ii) injection of B16-LS9-luc^+^ cells in the embryo eye, followed by incubation of engrafted embryos with 10 µM paclitaxel dissolved in fish water—an experimental procedure frequently used to test compounds in zebrafish [39]; (iii) engraftment of cells in the zebrafish embryo eye, followed by injection of the drug at 0.4 pmoles/embryo in the same eye. At the end of each protocol, the growth of B16-LS9-luc^+^ grafts was assessed by bioluminescence-based quantification of luc^+^ tumor cells performed at t_4_.

As anticipated, pretreatment with paclitaxel resulted in a significant inhibition of the growth of the tumor grafts (Figure 4D). No inhibition of the growth of B16-LS9-luc^+^ grafts was observed when engrafted embryos were treated with paclitaxel dissolved in the fish water, possibly as a consequence of the limited entry of the drug in the eye compartment (Figure 4E). Interestingly, a significant inhibition of B16-LS9-luc^+^ tumor growth occurred when paclitaxel was directly injected in the embryo eye after cell grafting (Figure 4F).

**Figure 5:**
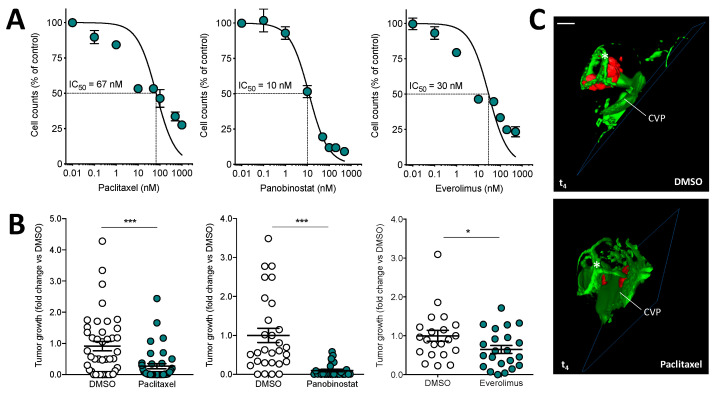
Effect of anticancer drugs on the growth of human uveal melanoma 92.1-RFP^+^/luc^+^ xenografts. (**A**) Effect of paclitaxel, panobinostat, and everolimus treatments on the proliferation of 92.1-RFP^+^/luc^+^ cells in vitro. Viable cells were counted after 48 h of incubation with increasing concentrations of paclitaxel or panobinostat or after 72 h of incubation with everolimus. Data are the mean ± SEM of two independent experiments. (**B**) After 92.1-RFP^+^/luc^+^ cell grafting into the zebrafish eye, 0.4 pmoles/embryo of paclitaxel, panobinostat, everolimus or the corresponding volume of DMSO were injected in the same eye. Tumor growth was evaluated at t_4_ by measuring the cell luminescence signal in the lysates of the whole embryos. Data are the mean ± SEM of two independent experiments. Each dot represents one embryo. * *p* < 0.05 and *** *p* < 0.001 vs. DMSO, Student’s *t*-test. (**C**) 3D reconstruction of the eye region of 92.1-RFP^+^/luc^+^ xenografts evaluated 4 days post implantation in the absence or in the presence of paclitaxel injection. Scale bar: 50 µm. Asterisk indicates the superficial ocular vasculature; CVP, choroidal vascular plexus.

Based on these observations, we decided to extend this experimental model by setting up an orthotopic experimental protocol in which human 92.1-RFP^+^/luc^+^ uveal melanoma cells were grafted (100 cells/embryo) in zebrafish embryo eyes at 48 hpf, followed by injection in the same eyes with 0.4 pmoles of different canonical chemotherapeutic drugs (i.e., paclitaxel [38], the histone deacetylase inhibitor panobinostat [40], the mTOR inhibitor everolimus [41], or vehicle). As shown in Figure 5A, all drugs inhibited the growth of uveal melanoma 92.1-RFP^+^/luc^+^ cells in vitro, with ID_50_ values ranging between 10 nM and 67 nM. Accordingly, treatment of engrafted uveal melanoma cells by eye injection of paclitaxel, panobinostat, or everolimus caused a significant inhibition of tumor growth when assessed by measurement of bioluminescence (Figure 5B). Notably, panobinostat exerts a pro-apoptotic effect on cancer cells, both in vitro and in vivo (Supplementary Materials, Figure S4A,B). Moreover, no significant toxic or pro-apoptotic effect was observed in the zebrafish embryo eye tissue after the injection of the three drugs (Supplementary Materials, Figure S4C).

In addition, light sheet fluorescence microscopy confirmed the efficacy of drug treatment, uveal melanoma cells remaining confined at the bottom of the eye in the proximity of the choroidal vasculature (Figure 5C). These results are in line with previous observations about the efficacy of these drugs on uveal melanoma growth in in vitro and in vivo experimental models [42,43]. Relevant to this point, it must be pointed out that phase 2 clinical trials designed to evaluate the clinical benefits of paclitaxel or everolimus administration showed only a limited efficacy in uveal melanoma metastatic patients [44,45], whereas no data are available about the effect of panobinostat. Further studies will be required to assess the efficacy of histone deacetylase inhibitors in uveal melanoma.

In this paper, we describe the first orthotopic model of uveal melanoma in zebrafish, previous models of uveal melanoma being limited to the injection of cancer cells into the yolk sac of embryos [46,47,48,49]. Even though orthotopic models are usually less rapid and more technically challenging with respect to the heterotopic implants, these approaches are more tissue-specific and allow a more realistic recapitulation of the natural microenvironment in which the tumor originated. Altogether, our data extend previous observations about the possibility of engrafting tumor cells, including retinoblastoma and conjunctival melanoma cells, in zebrafish embryo eyes, thus generating orthotopic models of different ocular neoplasms [7,8,9]. In addition, it should be considered that the eye represents a metastatic site for various tumor types, including cutaneous melanoma, breast, and lung cancer, with choroidal metastases occurring in approximately 8% of human malignancies [50]. Thus, tumor cell grafting in the zebrafish embryo eye may be exploited as a useful orthotopic model to investigate novel therapeutic approaches not only for primary tumors but for eye metastases as well. Relevant to this point, our work focuses on providing a simple and reliable strategy for the accurate quantification of engrafted tumor cells by exploiting the bioluminescent signal of firefly luciferase-expressing cells. Indeed, the presence of the lens and the cup-like structure of the eye make difficult the acquisition of high-quality fluorescent images and may lead to misleading results. Moreover, the autofluorescent properties of zebrafish embryos and mammalian cells increase the non-specific background and decrease the sensitivity of signal detection. On the other hand, bioluminescence displays a higher detection capacity and allows for greater sensitivity because of the enzymatic nature of the bioluminescent reporter and the absence of the endogenous bioluminescence of cellular components.

The luminescence-based method herein described allows for a precise quantification without relying on any image analysis software and it provides a simple and quick in vivo evaluation of the efficacy of anticancer drugs after intraocular delivery. In this context, this approach may be exploited for high-throughput analysis and may have relevant implications for the evaluation of new low molecular weight compounds for the treatment of uveal melanoma and other primary ocular neoplasms and metastatic tumors endowed with ocular tropism.

## 4. Conclusions

In this paper, we described an orthotopic model of uveal melanoma in which tumor cells are grafted in the eye of zebrafish embryos in the proximity of the developing choroidal vasculature. In the following 3–4 days, grafted cells proliferate and move towards the eye surface, thus demonstrating that the zebrafish embryo eye is a permissive environment for the growth of UM cells. In addition, the use of firefly luciferase bioluminescent murine and human tumor cells allowed the assessment of the antitumor activity of candidate drugs when injected into the grafted eyes. In conclusion, we have established a new quantification method based on the ocular implantation of bioluminescent uveal melanoma cells in zebrafish embryos that may represent a useful in vivo orthotopic model suitable for the screening of novel drug candidates for uveal melanoma therapy.

## Data Availability

Data are contained within the article or the Supplementary Materials.

## Supplementary Materials

The following are available online at https://www.mdpi.com/article/10.3390/biomedicines9121873/s1, Figure S1: Fluorescence-based quantification of the growth of murine melanoma B16-BL6-DsRed^+^ xenografts, Figure S2: Luciferase-based quantification of human melanoma A375M-RFP^+^/luc^+^ xenografts, Figure S3: Luciferase-based quantification of murine melanoma B16-LS9-luc^+^ xenografts, Figure S4: Everolimus, paclitaxel, and panobinostat effects on cell apoptosis.

## References

[1] Santoriello C., Zon L.I. (2012). Hooked! Modeling human disease in zebrafish. J. Clin. Investig..

[2] Tobia C., Gariano G., De Sena G., Presta M. (2013). Zebrafish embryo as a tool to study tumor/endothelial cell cross-talk. Biochim. Biophys. Acta.

[3] Rezzola S., Paganini G., Semeraro F., Presta M., Tobia C. (2016). Zebrafish (*Danio rerio*) embryo as a platform for the identification of novel angiogenesis inhibitors of retinal vascular diseases. Biochim. Biophys. Acta.

[4] Lee H.-C., Lin C.-Y., Tsai H.-J. (2021). Zebrafish, an in vivo platform to screen drugs and proteins for biomedical use. Pharmaceuticals.

[5] Barriuso J., Nagaraju R., Hurlstone A. (2015). Zebrafish: A new companion for translational research in oncology. Clin. Cancer Res..

[6] Letrado P., De Miguel I., Lamberto I., Díez-Martínez R., Oyarzabal J. (2018). Zebrafish: Speeding up the cancer drug discovery process. Cancer Res..

[7] Jo D.H., Son D., Na Y., Jang M., Choi J.-H., Kim J.H., Yu Y.S., Seok S.H., Kim J.H. (2013). Orthotopic transplantation of retinoblastoma cells into vitreous cavity of zebrafish for screening of anticancer drugs. Mol. Cancer.

[8] Chen X., Wang J., Cao Z., Hosaka K., Jensen L., Yang H., Sun Y., Zhuang R., Liu Y., Cao Y. (2015). Invasiveness and metastasis of retinoblastoma in an orthotopic zebrafish tumor model. Sci. Rep..

[9] Chen Q., Ramu V., Aydar Y., Groenewoud A., Zhou X.-Q., Jager M.J., Cole H., Cameron C.G., McFarland S.A., Bonnet S. (2020). TLD1433 photosensitizer inhibits conjunctival melanoma cells in zebrafish ectopic and orthotopic tumour models. Cancers.

[10] Zhang B., Shimada Y., Kuroyanagi J., Umemoto N., Nishimura Y., Tanaka T. (2014). Quantitative phenotyping-based in vivo chemical screening in a zebrafish model of leukemia stem cell xenotransplantation. PLoS ONE.

[11] Hill D., Chen L., Snaar-Jagalska E., Chaudhry B. (2018). Embryonic zebrafish xenograft assay of human cancer metastasis. F1000Research.

[12] Jovanovic P., Mihajlovic M., Djordjevic-Jocic J., Vlajkovic S., Cekic S., Stefanovic V. (2013). Ocular melanoma: An overview of the current status. Int. J. Clin. Exp. Pathol..

[13] Yonekawa Y., Kim I.K. (2012). Epidemiology and management of uveal melanoma. Hematol. Oncol. Clin. N. Am..

[14] Mahendraraj K., Lau C.S., Lee I., Chamberlain R.S. (2016). Trends in incidence, survival, and management of uveal melanoma: A population-based study of 7516 patients from the Surveillance, Epidemiology, and End Results database (1973–2012). Clin. Ophthalmol..

[15] Vivet-Noguer R., Tarin M., Roman-Roman S., Alsafadi S. (2019). Emerging therapeutic opportunities based on current knowledge of uveal melanoma biology. Cancers.

[16] Bedikian A.Y. (2006). Metastatic uveal melanoma therapy. Int. Ophthalmol. Clin..

[17] Croce M., Ferrini S., Pfeffer U., Gangemi R. (2019). Targeted therapy of uveal melanoma: Recent failures and new perspectives. Cancers.

[18] Diaz C.E., Rusciano D., Dithmar S., Grossniklaus H.E. (1999). B16LS9 melanoma cells spread to the liver from the murine ocular posterior compartment (PC). Curr. Eye Res..

[19] Ronca R., Giacomini A., Di Salle E., Coltrini D., Pagano K., Ragona L., Matarazzo S., Rezzola S., Maiolo D., Torella R. (2015). Long-pentraxin 3 derivative as a small-molecule FGF trap for cancer therapy. Cancer Cell.

[20] De Waard-Siebinga I., Blom D.-J.R., Griffioen M., Schrier P.I., Hoogendoorn E., Beverstock G., Danen E.H.J., Jager M.J. (1995). Establishment and characterization of an uveal-melanoma cell line. Int. J. Cancer.

[21] Rezzola S., Guerra J., Chandran A.M.K., Loda A., Cancarini A., Sacristani P., Semeraro F., Presta M. (2021). VEGF-independent activation of Müller cells by the vitreous from proliferative diabetic retinopathy patients. Int. J. Mol. Sci..

[22] Westerfield M. (2000). The Zebrafish Book. A Guide for the Laboratory Use of Zebrafish (Danio rerio).

[23] Kimmel C.B., Ballard W.W., Kimmel S.R., Ullmann B., Schilling T.F. (1995). Stages of embryonic development of the zebrafish. Dev. Dyn..

[24] Schindelin J., Arganda-Carreras I., Frise E., Kaynig V., Longair M., Pietzsch T., Preibisch S., Rueden C., Saalfeld S., Schmid B. (2012). Fiji: An open-source platform for biological-image analysis. Nat. Methods.

[25] Sabaliauskas N.A., Foutz C.A., Mest J.R., Budgeon L.R., Sidor A.T., Gershenson J.A., Joshi S.B., Cheng K.C. (2006). High-throughput zebrafish histology. Methods.

[26] Malicki J., Neuhauss S.C., Schier A.F., Solnica-Krezel L., Stemple D.L., Stainier D.Y., Abdelilah S., Zwartkruis F., Rangini Z., Driever W. (1996). Mutations affecting development of the zebrafish retina. Development.

[27] Hashiura T., Kimura E., Fujisawa S., Oikawa S., Nonaka S., Kurosaka D., Hitomi J. (2017). Live imaging of primary ocular vasculature formation in zebrafish. PLoS ONE.

[28] Singh A., Nüsslein-Volhard C. (2015). Zebrafish stripes as a model for vertebrate colour pattern formation. Curr. Biol..

[29] Antinucci P., Hindges R. (2016). A crystal-clear zebrafish for in vivo imaging. Sci. Rep..

[30] Rusciano D., Lorenzoni P., Burger M. (1999). Regulation of c-met expression in B16 murine melanoma cells by melanocyte stimulating hormone. J. Cell Sci..

[31] Elia G., Ren Y., Lorenzoni P., Zarnegar R., Burger M.M., Rusciano D. (2001). Mechanisms regulating c-met overexpression in liver-metastatic B16-LS9 melanoma cells. J. Cell. Biochem..

[32] Jones N.M., Yang H., Zhang Q., Morales-Tirado V.M., Grossniklaus H.E. (2019). Natural killer cells and pigment epithelial-derived factor control the infiltrative and nodular growth of hepatic metastases in an Orthotopic murine model of ocular melanoma. BMC Cancer.

[33] Stei M.M., Loeffler K.U., Holz F.G., Herwig-Carl M. (2016). Animal models of uveal melanoma: Methods, applicability, and limitations. BioMed Res. Int..

[34] Yang W., Li H., Mayhew E., Mellon J., Chen P.W., Niederkorn J.Y. (2011). NKT cell exacerbation of liver metastases arising from melanomas transplanted into either the eyes or spleens of mice. Investig. Opthalmol. Vis. Sci..

[35] Yang H., Brackett C.M., Morales-Tirado V.M., Li Z., Zhang Q., Wilson M.W., Benjamin C., Harris W., Waller E.K., Gudkov A. (2016). The toll-like receptor 5 agonist entolimod suppresses hepatic metastases in a murine model of ocular melanoma via an NK cell-dependent mechanism. Oncotarget.

[36] Ashur-Fabian O., Zloto O., Fabian I., Tsarfaty G., Ellis M., Steinberg D.M., Hercbergs A., Davis P.J., Fabian I.D. (2018). Tetrac delayed the onset of ocular melanoma in an orthotopic mouse model. Front. Endocrinol..

[37] Rezzola S., Ronca R., Loda A., Nawaz M.I., Tobia C., Paganini G., Maccarinelli F., Giacomini A., Semeraro F., Mor M. (2019). The autocrine FGF/FGFR system in both skin and uveal melanoma: FGF trapping as a possible therapeutic approach. Cancers.

[38] Owinsky E.R.K.R., Onehower R.O.C.D. (1995). Paclitaxel (taxol). N. Engl. J. Med..

[39] Cassar S., Adatto I., Freeman J.L., Gamse J.T., Iturria I., Lawrence C., Muriana A., Peterson R.T., Van Cruchten S., Zon L.I. (2020). Use of zebrafish in drug discovery toxicology. Chem. Res. Toxicol..

[40] Scuto A., Kirschbaum M., Kowolik C., Kretzner L., Juhasz A., Atadja P., Pullarkat V., Bhatia R., Forman S., Yen Y. (2008). The novel histone deacetylase inhibitor, LBH589, induces expression of DNA damage response genes and apoptosis in Ph– acute lymphoblastic leukemia cells. Blood.

[41] O’Reilly T., McSheehy P.M. (2010). Biomarker development for the clinical activity of the mTOR inhibitor everolimus (RAD001): Processes, limitations, and further proposals. Transl. Oncol..

[42] Faião-Flores F., Emmons M.F., Durante M.A., Kinose F., Saha B., Fang B., Koomen J.M., Chellappan S.P., Maria-Engler S., Rix U. (2019). HDAC inhibition enhances the in vivo efficacy of MEK inhibitor therapy in uveal melanoma. Clin. Cancer Res..

[43] Amirouchene-Angelozzi N., Frisch-Dit-Leitz E., Carita G., Dahmani A., Raymondie C., Liot G., Gentien D., Némati F., Decaudin D., Roman-Roman S. (2016). The mTOR inhibitor Everolimus synergizes with the PI3K inhibitor GDC0941 to enhance anti-tumor efficacy in uveal melanoma. Oncotarget.

[44] Shoushtari A.N., Ong L.T., Schoder H., Singh-Kandah S., Abbate K.T., Postow M.A., Callahan M.K., Wolchok J., Chapman P.B., Panageas K.S. (2016). A phase 2 trial of everolimus and pasireotide long-acting release in patients with metastatic uveal melanoma. Melanoma Res..

[45] Homsi J., Bedikian A.Y., Papadopoulos N.E., Kim K.B., Hwu W.-J., Mahoney S.L., Hwu P. (2010). Phase 2 open-label study of weekly docosahexaenoic acid–paclitaxel in patients with metastatic uveal melanoma. Melanoma Res..

[46] Van der Ent W., Burrello C., Teunisse A.F.A.S., Ksander B.R., Van Der Velden P.A., Jager M.J., Jochemsen A.G., Snaar-Jagalska B.E. (2014). Modeling of human uveal melanoma in zebrafish xenograft embryos. Investig. Opthalmol. Vis. Sci..

[47] Fornabaio G., Barnhill R.L., Lugassy C., Bentolila L.A., Cassoux N., Roman-Roman S., Alsafadi S., Del Bene F. (2018). Angiotropism and extravascular migratory metastasis in cutaneous and uveal melanoma progression in a zebrafish model. Sci. Rep..

[48] Van der Ent W., Burrello C., De Lange M.J., Van Der Velden P.A., Jochemsen A.G., Jager M.J., Snaar-Jagalska B.E. (2015). Embryonic zebrafish: Different phenotypes after injection of human uveal melanoma cells. Ocul. Oncol. Pathol..

[49] Yu L., Zhou D., Zhang G., Ren Z., Luo X., Liu P., Plouffe S.W., Meng Z., Moroishi T., Li Y. (2021). Co-occurrence of BAP1 and SF3B1 mutations in uveal melanoma induces cellular senescence. Mol. Oncol..

[50] Arepalli S., Kaliki S., Shields C.L. (2015). Choroidal metastases: Origin, features, and therapy. Indian J. Ophthalmol..

