# Supplementary material for: An Orthotopic Model of Uveal Melanoma in Zebrafish Embryo: A Novel Platform for Drug Evaluation
Source: Biomedicines. 2021 Dec 10;9(12):1873. doi: 10.3390/biomedicines9121873 (PMC8698893; doi:10.3390/biomedicines9121873)
Supplement: Supplementary file 1 [file biomedicines-09-01873-s001.zip › biomedicines-1405424-supplementary.pdf]

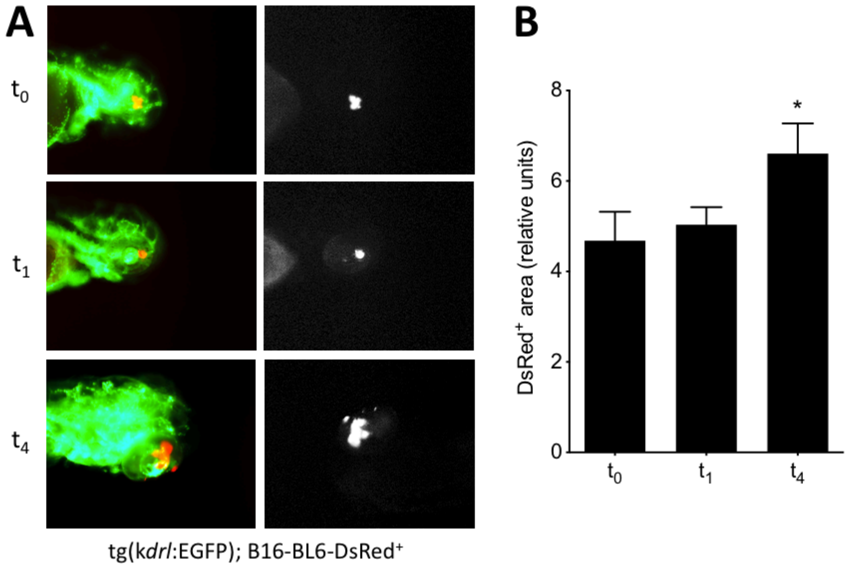

Figure S1. Fluorescence-based quantification of the growth of murine melanoma B16-BL6-DsRed+ xenografts. Murine melanoma B16-BL6-DsRed+ cells (100 cells/embryo) were injected in the posterior side of the developing eye of transgenic Tg(*kdrl*:EGFP) zebrafish embryos at 48 hpf. **A)** Maximum intensity projection of the z-stacks of B16-BL6-DsRed+ cell grafts assessed at 1 h (t_0), 1 day (t_1) and 4 days (t_4) post implantation. **B)** DsRed+ tumor area was quantified by FIJI software at t_0, t_1 and t_4 post implantation (n=8). $^*p < 0.05$ *vs* t_0, ANOVA.

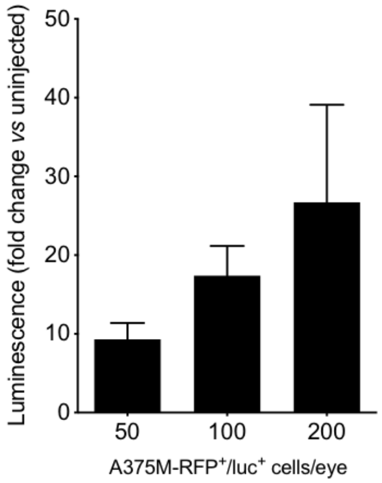

Figure S2. Luciferase-based quantification of human melanoma A375M-RFP+/luc+ xenografts. Human melanoma A375M-RFP+/luc+ cells (50, 100, and 200 cells/embryo) were injected in the posterior side of the developing eye of transgenic Tg(*kdrl*:EGFP) zebrafish embryos at 48 hpf. One h after grafting, eyes were enucleated and A375M-RFP+/luc+ bioluminescence signal was quantified and compared to basal levels measured in non-injected contralateral eyes.

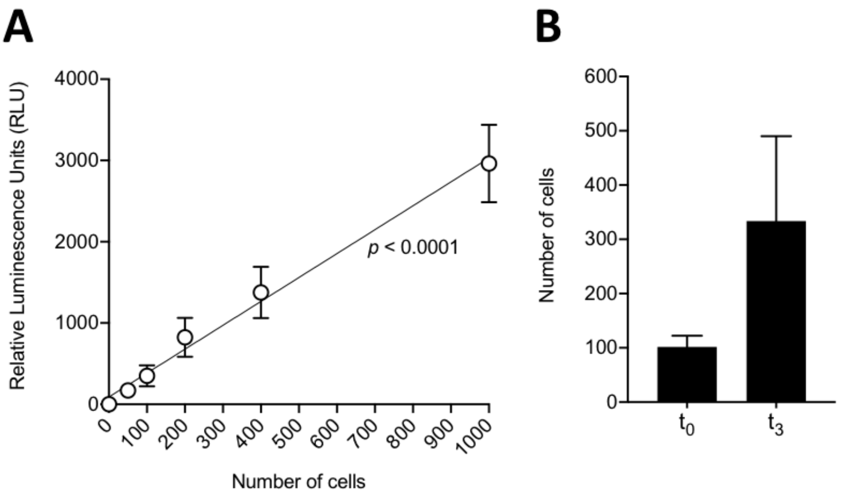

Figure S3. Luciferase-based quantification of murine melanoma B16-LS9-luc$^+$ xenografts. **(A)** Murine melanoma B16-LS9-luc$^+$ cells (0, 50, 100, 200, 400, and 1000 cells) were added to non-injected embryos and then the bioluminescence signal in the lysates was quantified. The values, subtracted of the blank, were plotted to obtain a calibration curve. **(B)** B16-LS9-luc$^+$ cells were injected in the posterior side of the developing eye of transgenic Tg(*kdrl*:EGFP) zebrafish embryos at 48 hpf. The cell luminescence signal was quantified at t_0 and t_3 in the lysates of the whole embryos and the number of cells extrapolated from the calibration curve. Data are mean ± SD (n=21 and 26 for t_0 and t_3, respectively).

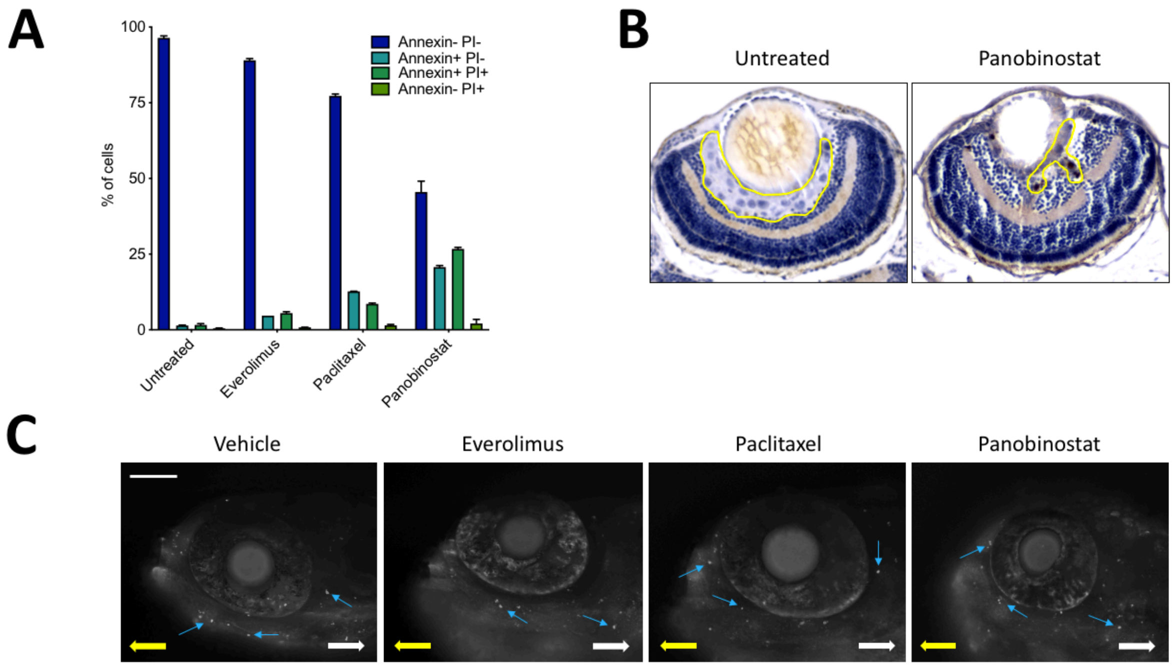

Figure S4. Everolimus, paclitaxel and panobinostat effect on cell apoptosis. (**A**) Cytofluorimetric analysis of apoptosis induced in 92.1-RFP$^+$/luc$^+$ cells after 72 h of treatment with 60 nM everolimus, 140 nM paclitaxel, or 20 nM panobinostat. (**B**) Immunohistochemical analysis of zebrafish embryo eyes at 4 days (t_4) after orthotopic injection of 92.1-RFP$^+$/luc$^+$ cells. Cleaved caspase 3 is detected in brown. Tumor area is highlighted in yellow. **(C)** 0.4 pmoles/embryo of everolimus, paclitaxel, panobinostat or the corresponding volume of DMSO were injected in the eye of zebrafish embryos. Apoptosis was evaluated at t_4 by acridine orange staining. White and yellow arrows indicate embryo orientation: white arrow, posterior side; yellow arrow, anterior side. Blue arrows show some representative zebrafish embryo apoptotic cells positive to the staining. Scale bar: 100 µm.
